# Supplementary material for: End Sequence Analysis Toolkit (ESAT) expands the extractable information from single-cell RNA-seq data
Source: Genome Res. 2016 Oct;26(10):1397–410. doi: 10.1101/gr.207902.116 (PMC5052061; doi:10.1101/gr.207902.116)
Supplement: Supplemental Material [file supp_gr.207902.116_Supp_Materials.tar.gz › ESAT-esat_v0.1/doc/html/classbroad_1_1core_1_1annotation_1_1_short_b_e_d.html]

ESAT: broad.core.annotation.ShortBED Class Reference


ESAT


- Main Page
- Related Pages
- Packages
- Classes
- Files

- Class List
- Class Index
- Class Hierarchy
- Class Members

All Classes Namespaces Files Functions Variables Enumerator Pages

- broad
- core
- annotation
- ShortBED

Public Member Functions |
List of all members

broad.core.annotation.ShortBED Class Reference

Inheritance diagram for broad.core.annotation.ShortBED:

[legend]

Collaboration diagram for broad.core.annotation.ShortBED:

[legend]

|  |  |
| --- | --- |
| Public Member Functions | |
|  | ShortBED (String name) |
|  | |
|  | ShortBED (LightweightGenomicAnnotation anot) |
|  | |
|  | ShortBED (String name, String chr, int start, int end) |
|  | |
|  | ShortBED (String[] info) throws ParseException |
|  | |
| String | toString () |
|  | |
| String | toString (boolean setNegativeScoresTo0) |
|  | |
| String | toShortString () |
|  | |
| String | toWIGString () |
|  | |
| Public Member Functions inherited from broad.core.annotation.BasicGenomicAnnotation | |
|  | BasicGenomicAnnotation (String name) |
|  | |
|  | BasicGenomicAnnotation (String name, String chr, int start, int end) |
|  | |
|  | BasicGenomicAnnotation (String name, String chr, int start, int end, Strand strand) |
|  | |
|  | BasicGenomicAnnotation (LightweightGenomicAnnotation ga) |
|  | |
|  | BasicGenomicAnnotation (Annotation ga) |
|  | |
|  | BasicGenomicAnnotation (GenomicAnnotation ga) |
|  | |
|  | BasicGenomicAnnotation (String[] info) |
|  | |
| int | getLength () |
|  | |
| String | toString () |
|  | |
| String | getId () |
|  | |
| void | setId (String id) |
|  | |
| void | reduceToDifference (GenomicAnnotation other) |
|  | |
| List< Annotation > | disect (Annotation a) |
|  | |
| List< Annotation > | disect (List<?extends Annotation > disectors) |
|  | |
| boolean | isFlankedBy (TwoSubjectAnnotation twoSubjectAnnotation, int buffer) |
|  | |
| int | lastIdxWhereListSearchBroke () |
|  | |
| Sequence | getSequence () |
|  | |
| void | setSequence (Sequence sequence) |
|  | |
| void | setOrientation (boolean orientation) |
|  | |
| void | setThreePrimeBuffer (int bufferSize) |
|  | |
| void | setFivePrimeBuffer (int bufferSize) |
|  | |
| int | getFivePrimeBases () |
|  | |
| int | getThreePrimeBases () |
|  | |
| int | compareTo (GenomicAnnotation arg0) |
|  | |
| int | getOrientedEnd () |
|  | |
| int | getOrientedStart () |
|  | |
| void | addBlock (String name, int start, int end) |
|  | |
| boolean | mayHaveBlocks () |
|  | |
| Public Member Functions inherited from broad.core.annotation.BasicLightweightAnnotation | |
|  | BasicLightweightAnnotation () |
|  | |
|  | BasicLightweightAnnotation (String chr, int start, int end) |
|  | |
|  | BasicLightweightAnnotation (String chr, int start, int end, String orientation) |
|  | |
|  | BasicLightweightAnnotation (String chr, int start, int end, Strand orientation, String name) |
|  | |
|  | BasicLightweightAnnotation (String chr, int start, int end, String orientation, double Scr) |
|  | |
|  | BasicLightweightAnnotation (String chr, String start, String end) |
|  | |
|  | BasicLightweightAnnotation (LightweightGenomicAnnotation annotation) |
|  | |
|  | BasicLightweightAnnotation (Annotation annotation) |
|  | |
| String | getChromosomeString () |
|  | |
| int | getDistanceTo (LightweightGenomicAnnotation other) |
|  | |
| double | getExtraScore (int i) |
|  | |
| List< Double > | getExtraScores () |
|  | |
| String | getLocationString () |
|  | |
| long | getMiddle () |
|  | |
| int | getOverlap (LightweightGenomicAnnotation other) |
|  | |
| double | getScore () |
|  | |
| void | setReversedOrientation (boolean isInReversedOrientation) |
|  | |
| void | setScore (double score) |
|  | |
| boolean | almostEqual (LightweightGenomicAnnotation other, int fudge) |
|  | |
| boolean | contains (LightweightGenomicAnnotation other) |
|  | |
| boolean | inReversedOrientation () |
|  | |
| boolean | overlaps (Collection<?extends Annotation > others, int buffer) |
|  | |
| boolean | overlaps (Collection<?extends Annotation > others) |
|  | |
| void | addExtraScore (double score) |
|  | |
| void | removeExtraScores () |
|  | |
| void | setBoundariesFromAnnoations (List<?extends GenomicAnnotation > annotations) |
|  | |
| void | stitchTo (LightweightGenomicAnnotation other) |
|  | |
| void | takeIntersection (LightweightGenomicAnnotation other) |
|  | |
| void | takeUnion (LightweightGenomicAnnotation other) |
|  | |
| LightweightGenomicAnnotation | intersect (LightweightGenomicAnnotation other) |
|  | |
| Collection<?extends Annotation > | intersect (Collection<?extends Annotation > annotations) |
|  | |
| int | hashCode () |
|  | |
| int | length () |
|  | |
| String | toUCSC () |
|  | |
| String | toString () |
|  | |
| int | compareTo (LightweightGenomicAnnotation b) |
|  | |
| void | setOrientation (String orientation) |
|  | |
| String | getChromosome () |
|  | |
| void | setChromosome (String chr) |
|  | |
| Public Member Functions inherited from broad.core.annotation.LightweightGenomicAnnotation | |
| int | getStart () |
|  | |
| int | getEnd () |
|  | |
| String | getName () |
|  | |
| void | setName (String name) |
|  | |
| void | setStart (int start) |
|  | |
| void | setEnd (int end) |
|  | |
| Public Member Functions inherited from umms.core.annotation.Annotation | |
| int | getSAMStart () |
|  | |
| int | getSAMEnd () |
|  | |
| String | getReferenceName () |
|  | |
| String | getChr () |
|  | |
| Strand | getStrand () |
|  | |
| boolean | hasOrientation () |
|  | |
| boolean | isNegativeStrand () |
|  | |
| int | numBlocks () |
|  | |
| List<?extends Annotation > | getBlocks (boolean oriented) |
|  | |
| int | getSize () |
|  | |
| int | size () |
|  | |
| int | getLengthOnReference () |
|  | |
| boolean | isUnoriented () |
|  | |
| int | getReferenceCoordinateAtPosition (int positionInAnnotation) |
|  | |
| int | getPositionAtReferenceCoordinate (int referenceCoordinate) |
|  | |
| int | getReferenceCoordinateAtPosition (int positionInAnnotation, boolean ignoreOrientation) |
|  | |
| int | getPositionAtReferenceCoordinate (int referenceCoordinate, boolean ignoreOrientation) |
|  | |
| void | setOrientation (char orientation) |
|  | |
| void | setOrientation (Strand orientation) |
|  | |
| void | setOrientedStart (int orientedStart) |
|  | |
| void | setOrientedEnd (int orientedEnd) |
|  | |
| void | setReferenceName (String refName) |
|  | |
| boolean | equals (Annotation other) |
|  | |
| void | expand (int deltaStart, int deltaEnd) |
|  | |
| Annotation | trim (int deltaStart, int deltaEnd) |
|  | |
| void | shift (int delta) |
|  | |
| void | moveToCoordinate (int coordinateInReference) |
|  | |
| Annotation | copy () |
|  | |
| Annotation | minus (Annotation other) |
|  | |
| Annotation | minus (Collection<?extends Annotation > others) |
|  | |
| int | getDistanceTo (Annotation other) |
|  | |
| String | toBED () |
|  | |
| String | toBED (int r, int g, int b) |
|  | |
| String | toShortBED () |
|  | |
| String | toBEDGraph () |
|  | |
| String | getFullInfoString () |
|  | |
| boolean | overlaps (Annotation other, int buffer) |
|  | |
| boolean | overlaps (Annotation other) |
|  | |
| boolean | overlaps (Annotation other, int buffer, boolean considerOrientation) |
|  | |
| boolean | overlaps (Annotation other, boolean considerOrientation) |
|  | |
| boolean | overlapsStranded (Annotation other) |
|  | |
| int | getOverlap (Annotation other) |
|  | |
| boolean | contains (Annotation other) |
|  | |
| Annotation | union (Annotation other) |
|  | |
| Annotation | intersect (Annotation other) |
|  | |
| List< Annotation > | intersect (List<?extends Annotation > annotations) |
|  | |
| int | compareToAnnotation (Annotation b) |
|  | |
| void | stitchTo (Annotation next) |
|  | |
| String | toCigar () |
|  | |
| boolean | fullyContains (Annotation annotation) |
|  | |
| Annotation | complement () |
|  | |
| boolean | equals (Annotation other, boolean useOrientation) |
|  | |
| Collection<?extends Annotation > | getSpliceConnections () |
|  | |
| int | getMidpoint () |
|  | |
| Public Member Functions inherited from broad.core.annotation.GenomicAnnotation | |
| void | setReversedOrientation (boolean isInReversedOrientation) |
|  | |
| void | setOrientation (String orientationString) |
|  | |
| Strand | getOrientation () |
|  | |
| String | getLocationString () |
|  | |
| List<?extends Annotation > | getBlocks () |
|  | |
| Public Member Functions inherited from broad.core.annotation.Feature | |
| String | getName () |
|  | |
| double | getScore () |
|  | |

|  |  |
| --- | --- |
| Additional Inherited Members | |
| Static Public Member Functions inherited from broad.core.annotation.BasicLightweightAnnotation | |
| static Collection  < BasicLightweightAnnotation > | mergeAllOverlappers (TreeSet< BasicLightweightAnnotation > regions) |
|  | |
| static LightweightGenomicAnnotation | createFromUCSC (String ucsc) |
|  | |
| static List< Annotation > | stitchList (Collection<?extends Annotation > sortedList, int maxDistanceToStitch) |
|  | |
| Protected Member Functions inherited from broad.core.annotation.BasicGenomicAnnotation | |
|  | BasicGenomicAnnotation () |
|  | |
| Protected Member Functions inherited from broad.core.annotation.BasicLightweightAnnotation | |
| void | setStart (String data) |
|  | |

## Constructor & Destructor Documentation

|  |  |  |  |  |  |
| --- | --- | --- | --- | --- | --- |
| broad.core.annotation.ShortBED.ShortBED | ( | String | *name* | ) |  |

|  |  |  |  |  |  |
| --- | --- | --- | --- | --- | --- |
| broad.core.annotation.ShortBED.ShortBED | ( | LightweightGenomicAnnotation | *anot* | ) |  |

|  |  |  |  |
| --- | --- | --- | --- |
| broad.core.annotation.ShortBED.ShortBED | ( | String | *name*, |
|  |  | String | *chr*, |
|  |  | int | *start*, |
|  |  | int | *end* |
|  | ) |  |  |

|  |  |  |  |  |  |
| --- | --- | --- | --- | --- | --- |
| broad.core.annotation.ShortBED.ShortBED | ( | String[] | *info* | ) | throws ParseException |

Here is the call graph for this function:

## Member Function Documentation

|  |  |  |  |  |
| --- | --- | --- | --- | --- |
| String broad.core.annotation.ShortBED.toShortString | ( |  | ) |  |

Here is the call graph for this function:

|  |  |  |  |  |
| --- | --- | --- | --- | --- |
| String broad.core.annotation.ShortBED.toString | ( |  | ) |  |

Here is the call graph for this function:

Here is the caller graph for this function:

|  |  |  |  |  |  |
| --- | --- | --- | --- | --- | --- |
| String broad.core.annotation.ShortBED.toString | ( | boolean | *setNegativeScoresTo0* | ) |  |

Here is the call graph for this function:

|  |  |  |  |  |
| --- | --- | --- | --- | --- |
| String broad.core.annotation.ShortBED.toWIGString | ( |  | ) |  |

Here is the call graph for this function:

---

The documentation for this class was generated from the following file:

- java/broad/core/annotation/ShortBED.java


---

Generated on Fri Aug 15 2014 15:10:51 for ESAT by  

 1.8.7
